# Fatty acid content and composition in edible *Ruspolia differens* feeding on mixtures of natural food plants

**DOI:** 10.1186/s13104-018-3792-9

**Published:** 2018-10-01

**Authors:** Karlmax Rutaro, Geoffrey M. Malinga, Vilma J. Lehtovaara, Robert Opoke, Philip Nyeko, Heikki Roininen, Anu Valtonen

**Affiliations:** 1 Department of Biochemistry and Sports Science, Makerere University, P.O. Box 7062, Kampala, Uganda; 2 Department of Environmental and Biological Sciences, University of Eastern Finland, P.O. Box 111, 80101 Joensuu, Finland; 3 Department of Biology, Gulu University, P.O. Box 166, Gulu, Uganda; 4 Department of Forestry, Biodiversity and Tourism, Makerere University, P.O. Box 7062, Kampala, Uganda

**Keywords:** Africa, Edible insect, Food security, Natural diets, MUFA, PUFA

## Abstract

**Objectives:**

To develop successful mass-rearing programs of edible insects, knowledge of the feeds and their influence on nutritional content is critical. We assessed the influence of natural food plants (grass inflorescences) and their mixtures on fatty acid profiles of edible *Ruspolia differens*. We reared neonate nymphs to adult on six dietary treatments consisting of one, and mixtures of two, three, five, six and eight plants.

**Results:**

The contents of saturated, monounsaturated and polyunsaturated fatty acids, omega-6/omega-3 ratio, and adult body weight did not differ among dietary treatments. However, the composition of fatty acids differed significantly among insects fed on six dietary treatments, but only for the rare fatty acids. Our results demonstrate that even if natural diets (grass inflorescences) do not strongly modify fatty acid contents or compositions of *R. differens*, when reared from neonate nymphs to adults, their n − 6/n − 3 fatty acid ratio is generally low and thus good for a healthy human diet.

## Introduction

*Ruspolia differens* (Tettigoniidae) is one of the most eaten insects in Sub-Saharan Africa with high nutritional and economic potential [1]. Currently, *R. differens* is harvested from the wild during the two annual swarming seasons. To ensure availability of *R. differens* throughout the year, inexpensive diets based on natural plants that could support the small-scale rearing in rural settings of Africa are urgently needed. In the wild, *R. differens* feeds mostly on the leaves, flowers and seeds of grass species, including cereal crops [2, 3], and their nutrient composition depends on the diet [4]. However, the effect of natural plant diets on the nutritional composition of *R. differens*, fatty acids in particular, needs to be investigated.

Here, we reared *R. differens*, from neonate nymphs to adult on six plant diets, to know how increasingly diversifying natural diets modify their body weight, and content and composition of their fatty acids. Specifically, we asked: Does the (i) body weight, (ii) content of saturated fatty acids (SFA), monounsaturated fatty acids (MUFA), polyunsaturated fatty acids (PUFA), (iii) n − 6/n − 3 fatty acid ratio, and (iv) fatty acid composition differ among individuals in the six dietary treatments, between the sexes or is there an interaction between diet and sex? We analyzed the adult insects, which are typically consumed [1, 5], in order to provide information that is comparable to previous studies [3, 4, 6].

## Main text

### Materials and methods

#### Study insects

The parent population was harvested around the Makerere University Agricultural Research Institute, Uganda. An equal number of sexes were transferred to plastic containers (24 cm length × 18 cm width × 12.5 cm height). The insects were fed ad libitum on *Panicum maximum.* Water was provided by inserting wet tissue paper. Females oviposited eggs to 5.3-cm wide × 7.1-cm high containers prefilled with wet cotton wool and sand. Eggs were incubated until hatching.

#### Diet treatments

The six dietary treatments comprised of one, and mixtures of two, three, five, six and eight grass species inflorescences (Table 1). The grass species were selected based on their acceptance by *R. differens* in preliminary feeding tests (Junes, unpubl. data).

**Table 1:** Composition of grass species inflorescence used in the six dietary treatments

Treatment name	Composition
One grass species inflorescence	Congo signal grass *Brachiaria ruziziensis* R. Germ. & C.M.Evrard
Two grass species inflorescence mixtures	Congo signal grass *Brachiaria ruziziensis* R.Germ. & C.M.Evrard Ribbon bristle grass *Setaria megaphylla* (Steud.) T.Durand & Schinz
Three grass species inflorescence mixtures	Congo signal grass *Brachiaria ruziziensis* R.Germ. & C.M.Evrard Ribbon bristle grass *Setaria megaphylla* (Steud.) T.Durand & Schinz Nandi grass *Setaria sphacelata*
Five grass species inflorescence mixtures	Congo signal grass *Brachiaria ruziziensis* R.Germ. & C.M.Evrard Ribbon bristle grass *Setaria megaphylla* (Steud.) T.Durand & Schinz Nandi grass *Setaria sphacelata* Antelope grass *Echinochloa pyramidalis* (Lam.) Hitchc. & Chase Elephant grass *Pennisetum purpureum* Schumach.
Six grass species inflorescence mixtures	Congo signal grass *Brachiaria ruziziensis* R.Germ. & C.M.Evrard Ribbon bristle grass *Setaria megaphylla* (Steud.) T.Durand & Schinz Nandi grass *Setaria sphacelata* Antelope grass *Echinochloa pyramidalis* (Lam.) Hitchc. & Chase Elephant grass *Pennisetum purpureum* Schumach. Rhodes grass *Chloris gayana* Kunth
Eight grass species inflorescence mixtures	Congo signal grass *Brachiaria ruziziensis* R.Germ. & C.M.Evrard Ribbon bristle grass *Setaria megaphylla* (Steud.) T.Durand & Schinz Nandi grass *Setaria sphacelata* Antelope grass *Echinochloa pyramidalis* (Lam.) Hitchc. & Chase Elephant grass *Pennisetum purpureum* Schumach. Rhodes grass *Chloris gayana* Kunth Wild finger millet *Eleusine indica* (L.) Gaertn Guinea grass *Panicum maximum* Jacq.

#### Experimental setup

The effect of the diet on weight, fatty acid content and composition of *R. differens* was evaluated by rearing 1–2 days old neonate nymphs to adult on the six dietary treatments (23–27 °C, 50–60% RH and 12L:12D photoperiod). Nymphs were individually reared in jars, covered with a netting cloth. The position of the jars in 10 blocks was routinely shuffled to cater for microclimatic variability. Approximately equal amounts of food (two florets) were given (i.e., the single-feed diet received two florets, the two-feed diet received one floret of each plant and so on), and only freshly opened inflorescences were used. The nymphs were fed ad libitum and water provided on wet tissue paper, and diets were replenished after every 3–4 days until moulting to adult. For emerged adults, we recorded the sex and the body weight. Sixty individuals were freeze-dried and five individuals per dietary treatment were randomly selected for fatty acid analyses.

#### Fatty acid analyses

Fatty acids were analysed at the Bio-Competence Centre for Healthy Dairy Products (Accreditation EN ISO/IEC 17025:2005), Tartu, Estonia, using direct transesterification [7], with minor modifications [4]. Fatty acid methyl esters were analysed on an Agilent 6890A GC, equipped with a FID detector and an auto sampler [4].

#### Statistical analyses

We fitted ANOVA models to analyze whether body weight, content of SFA, MUFA, PUFA, and n − 6/n − 3 fatty acid ratio of *R. differens* differed among the dietary treatments, sexes, or if there was an interaction between diet and sex. SFA, MUFA and n − 6/n − 3 were ln-transformed prior to analyses. We fitted PERMANOVA models (type III SS; 999 permutations) in PRIMER-E [8] to test if the composition of fatty acids differed among the dietary treatments, between sexes, and whether there was an interaction between diets and sex (using both untransformed and fourth-root transformed fatty acid proportions). A similarity percentage analysis in PRIMER-E [8] was run to explore which fatty acids contributed most to the dissimilarities. Permutational multivariate dispersion [9] test was used to test if the degree of variability in the relative proportions of fatty acids differed among dietary treatments.

### Results

#### Body weight

The body weight of individuals ranged from 0.41 g (least diversified) to 0.45 g (the most diversified diets) but did not differ among the dietary treatments (ANOVA; F_5, 18_ = 2.2, p = 0.098), between the sexes (F_1, 18_ = 2.8, p = 0.109), and there was no diet × sex interaction (F_5, 18_ = 0.8, p = 0.597).

#### Fatty acid content

The content of SFA did not differ between the six dietary treatments (ANOVA; F_5, 18_ = 0.1, p = 0.98), sexes (F_1, 18_ = 0.1, p = 0.82) and there was no diet × sex interaction (F_5, 18_ = 1.7, p = 0.18). MUFA content did not differ between the six dietary treatments (ANOVA; F_5, 18_ = 0.3, p = 0.89), sexes (F_1, 18_ = 0.001, p = 0.98) and there was no diet × sex interaction (F_5, 18_ = 1.7, p = 0.18). There was also no difference in the content of PUFA between the six dietary treatments (ANOVA; F_5, 18_ = 0.2, p = 0.96), sexes (F_1, 18_ = 0.1, p = 0.79) and no diet × sex interaction (F_5, 18_ = 1.4, p = 0.26). Finally, the sexes differed in the ratio of n − 6/n − 3 (F_1, 18_ = 4.7, p = 0.05), females having a lower n − 6/n − 3 ratio (mean = 1.6, SE = 0.2) than males (mean = 2.2, SE = 0.3). However, there was no difference in the ratio n − 6/n − 3 between the dietary treatments (ANOVA; F_5, 18_ = 0.6, p = 0.71) and no diet × sex interaction (F_5, 18_ = 1.9, p = 0.14).

#### Fatty acid composition

There were no differences in the composition of (untransformed) fatty acids (emphasizing the most common fatty acids) between the dietary treatments (PERMANOVA; pseudo-F_5, 18_ = 0.7, p = 0.66), sexes (pseudo-F_1, 18_ = 0.99, p = 0.35) and there was no diet × sex interaction (pseudo-F_5, 18_ = 1.69, p = 0.15). However, when the fatty acid compositions were fourth root transformed (emphasizing the rare fatty acids), the fatty acid compositions differed among the six dietary treatments (PERMANOVA; pseudo-F_5, 18_ = 3.3, p = 0.001), but not between the sexes (pseudo-F_1, 18_ = 1.4, p = 0.23) and there was no diet × sex interaction (pseudo-F_5, 18_ = 1.2, p = 0.29). The dietary treatment explained 33% of the variation in the fatty acid compositions. The differences in compositions were not explained by differing variation in fatty acid composition among diets (PERMDISP; F_5, 24_ = 0.6, p = 0.811). Based on the similarity percentage analysis, Eicosenoic (cis-11-eicosenoic) acid, gadoleic (cis-9-eicosenoic) acid, docosadienoic (cis-13, 16-docosadienoic) acid made the strongest contributions to the dissimilarities in the fatty acid composition across the dietary treatments (fourth-root transformed data). Eicosenoic and docosadienoic acids were more common in less diversified diets whereas gadoleic acid was more common in highly diversified diets (Table 2).

**Table 2:** Fatty acid proportions (%) of *R. differens* feeding on the six gradually diversifying natural diets consisting of one, and mixtures of two, three, five, six and eight grass species inflorescences

Fatty acid	Dietary treatment					
	One grass	Two grasses	Three grasses	Five grasses	Six grasses	Eight grasses
C10:0	0.00 ± 0.00	0.00 ± 0.00	0.00 ± 0.00	0.09 ± 0.09	0.00 ± 0.00	0.00 ± 0.00
C12:0	0.10 ± 0.01	0.04 ± 0.01	0.08 ± 0.01	0.1 ± 0.01	0.08 ± 0.01	0.06 ± 0.01
C14:0	0.75 ± 0.01	0.58 ± 0.09	0.68 ± 0.07	0.91 ± 0.18	0.81 ± 0.09	0.68 ± 0.15
C15:0	0.32 ± 0.05	0.29 ± 0.05	0.33 ± 0.05	0.38 ± 0.08	0.24 ± 0.05	0.23 ± 0.03
C16:0	20.74 ± 3.17	20.19 ± 2.05	19.61 ± 2.08	19.80 ± 3.71	22.26 ± 2.14	22.12 ± 3.49
C18:0	8.66 ± 0.90	8.80 ± 0.49	9.20 ± 0.83	9.09 ± 1.34	7.79 ± 0.81	7.69 ± 0.78
C20:0	1.27 ± 0.30	1.18 ± 0.25	1.41 ± 0.36	1.35 ± 0.29	0.94 ± 0.23	1.05 ± 0.29
C22:0	0.49 ± 0.14	0.44 ± 0.11	0.50 ± 0.13	0.51 ± 0.14	0.37 ± 0.14	0.36 ± 0.13
C23:0	0.03 ± 0.01	0.02 ± 0.01	0.01 ± 0.01	0.01 ± 0.01	0.03 ± 0.01	0.03 ± 0.02
C24:0	0.04 ± 0.01	0.05 ± 0.01	0.09 ± 0.02	0.07 ± 0.02	0.08 ± 0.01	0.08 ± 0.02
C26:0	0.24 ± 0.07	0.32 ± 0.21	0.17 ± 0.04	0.19 ± 0.04	0.20 ± 0.04	0.14 ± 0.05
∑SFA	32.62 ± 3.23	31.91 ± 1.47	32.10 ± 1.72	32.50 ± 3.93	32.80 ± 1.37	32.45 ± 3.81
C14:1n5t	0.00 ± 0.00	0.00 ± 0.00	0.02 ± 0.02	0.02 ± 0.02	0.01 ± 0.01	0.00 ± 0.00
C14:1n5	0.11 ± 0.05	0.08 ± 0.02	0.11 ± 0.03	0.11 ± 0.04	0.07 ± 0.01	0.09 ± 0.04
C16:1n9	0.07 ± 0.02	0.07 ± 0.01	0.1 ± 0.01	0.08 ± 0.02	0.09 ± 0.01	0.07 ± 0.00
C16:1n7	0.96 ± 0.16	0.76 ± 0.17	0.66 ± 0.12	0.85 ± 0.21	1.34 ± 0.40	1.15 ± 0.16
C17:1n10	0.17 ± 0.12	0.15 ± 0.05	0.27 ± 0.11	0.37 ± 0.10	0.32 ± 0.11	0.31 ± 0.10
C17:1n8	0.50 ± 0.13	0.46 ± 0.08	0.41 ± 0.15	0.42 ± 0.05	0.32 ± 0.02	0.27 ± 0.04
C18:1n10,12t	0.00 ± 0.00	0.00 ± 0.00	0.00 ± 0.00	0.00 ± 0.00	0.06 ± 0.03	0.03 ± 0.02
C18:1n9t	0.18 ± 0.03	0.18 ± 0.04	0.13 ± 0.05	0.19 ± 0.08	0.13 ± 0.04	0.08 ± 0.03
C18:1n7t	0.00 ± 0.00	0.00 ± 0.00	0.02 ± 0.02	0.00 ± 0.00	0.00 ± 0.00	0.00 ± 0.00
C18:1n9	20.46 ± 2.88	22.28 ± 2.81	19.51 ± 2.74	22.32 ± 3.18	27.25 ± 3.26	28.60 ± 2.30
C18:1n7	0.09 ± 0.02	0.10 ± 0.01	0.07 ± 0.02	0.09 ± 0.02	0.02 ± 0.01	0.00 ± 0.00
C20:1n11,12	0.00 ± 0.00	0.00 ± 0.00	0.00 ± 0.00	0.00 ± 0.00	0.19 ± 0.05	0.22 ± 0.07
C20:1n9	0.27 ± 0.07	0.23 ± 0.06	0.23 ± 0.04	0.28 ± 0.07	0.00 ± 0.00	0.00 ± 0.00
C22:1n9	0.00 ± 0.00	0.01 ± 0.01	0.00 ± 0.00	0.01 ± 0.01	0.00 ± 0.00	0.00 ± 0.00
C24:1n9	0.01 ± 0.01	0.02 ± 0.01	0.08 ± 0.07	0.01 ± 0.01	0.02 ± 0.01	0.02 ± 0.01
∑MUFA	22.81 ± 2.73	24.36 ± 2.74	21.61 ± 2.70	24.75 ± 3.15	29.83 ± 3.28	30.85 ± 2.16
C18:2n5c,9t	0.02 ± 0.00	0.02 ± 0.02	0.06 ± 0.06	0.05 ± 0.02	0.15 ± 0.06	0.06 ± 0.04
C18:2n6	26.27 ± 6.26	26.95 ± 2.65	28.45 ± 2.99	26.82 ± 6.01	21.03 ± 2.74	22.87 ± 5.16
C18:3n6	0.08 ± 0.02	0.05 ± 0.01	0.05 ± 0.02	0.08 ± 0.02	0.10 ± 0.02	0.08 ± 0.02
C18:3n3	16.34 ± 2.06	15.04 ± 2.26	15.88 ± 2.05	13.85 ± 1.35	14.72 ± 1.48	12.36 ± 1.23
CLA18:2n6t,8c	0.00 ± 0.00	0.02 ± 0.01	0.00 ± 0.00	0.00 ± 0.00	0.00 ± 0.00	0.00 ± 0.00
C20:2n6	0.00 ± 0.00	0.00 ± 0.00	0.00 ± 0.00	0.00 ± 0.00	0.09 ± 0.03	0.07 ± 0.03
C20:3n3	0.03 ± 0.01	0.04 ± 0.02	0.06 ± 0.06	0.03 ± 0.02	0.05 ± 0.02	0.01 ± 0.01
C22:2n6	0.16 ± 0.06	0.10 ± 0.06	0.01 ± 0.01	0.01 ± 0.01	0.04 ± 0.02	0.00 ± 0.00
C20:5n3	0.01 ± 0.01	0.00 ± 0.00	0.00 ± 0.00	0.00 ± 0.00	0.02 ± 0.01	0.06 ± 0.02
C22:4n6	0.01 ± 0.00	0.01 ± 0.01	0.02 ± 0.02	0.01 ± 0.01	0.02 ± 0.01	0.01 ± 0.00
C22:5n6	0.00 ± 0.00	0.00 ± 0.00	0.00 ± 0.00	0.02 ± 0.02	0.01 ± 0.01	0.04 ± 0.04
∑PUFA	42.90 ± 5.43	42.21 ± 3.94	44.43 ± 3.78	40.81 ± 6.09	36.08 ± 4.04	35.50 ± 5.42
n6/n3	1.85 ± 0.64	1.98 ± 0.41	1.89 ± 0.29	2.03 ± 0.53	1.45 ± 0.12	1.90 ± 0.44
iso/anteiso	0.19 ± 0.06	0.17 ± 0.03	0.26 ± 0.07	0.24 ± 0.07	0.22 ± 0.05	0.23 ± 0.07
C10:1n1c-C11:0	0.00 ± 0.00	0.00 ± 0.00	0.01 ± 0.01	0.00 ± 0.00	0.00 ± 0.00	0.00 ± 0.00
C18:1n3c + C19:0	1.43 ± 0.29	1.35 ± 0.31	1.46 ± 0.27	1.60 ± 0.42	0.87 ± 0.02	0.86 ± 0.24

Data are expressed as mean ± SE; n = 5; SFA, saturated fatty acids; MUFA, monounsaturated fatty acids; PUFA, polyunsaturated fatty acids; n6/n3, ratio of omega-6 to omega-3 fatty acids; C, number of carbon atoms in the fatty acid structure; c, *cis*; t, *trans* fatty acid; C10:1n1c-C11:0 and C18:1n3c + C19:0 were unresolved fatty acids, i.e., they were not separated during analysis and thus quantified together

Overall, the PUFAs contributed most to the fatty acid composition followed by MUFAs and SFAs (Table 2). The total PUFAs on dry weight basis ranged from 36 to 44% across diet treatments (Table 2). The most predominant PUFAs were linoleic acid (21–28%) and α-linolenic acid (12–16%) (Table 3). The proportion of SFAs ranged from 32 to 33% with palmitic acid (20–22%) and stearic acid (8–9%) being the most predominant fatty acids (Table 2). For MUFAs, the range was from 22 to 31% with oleic acid (20–29%) being the most predominant (Table 2).

**Table 3:** Percentage composition of the most common fatty acids in *R. differens* compared to previous studies (2010–2017)

Study	Common fatty acids					Fatty acid groups			Source
	C16:0	C18:0	C18:1n9	C18:2n6	C18:3n3	ƩSFA	ƩMUFA	ƩPUFA	
This study	19.6–22.3	7.7–9.2	19.5–28.6	21.0–28.5	12.4–16.3	31.9–32.8	21.6–30.9	35.5–44.4	Reared
Kinyuru et al. [6]	31.5–32.1	5.5–5.9	24.6–24.9	29.5–31.2	3.2–4.2	38.3–39.1	26.3–26.6	33.8–34.4	Wild harvest
Opio [17]	27.4–31.7	8.3–8.5	40.5–43.4	12.7–16.5	0.72–2.39	36.7–37.3	43.5–46.6	14.4–17.4	Wild harvest
Fombong et al. [5]	28.1–28.2	7.8–7.9	44.3–44.4	13.9–14.4	1.39–1.44	37.6–37.8	46.0–46.2	16.0–16.4	Wild
Lehtovaara et al. [4]	11.3–35.3	4.9–12.0	19.1–45.3	3.9–37.9	0.4–44.6	16.9–50.8	19.8–48.9	10.1–62.6	Reared

SFA: saturated fatty acid; C16:0-palmitic acid; C18:0-stearic acid; MUFA: Monounsaturated fatty acid-C18:1n9; PUFA: polyunsaturated fatty acid; C18:2n6-linoleic acid; C18:3n3: α-linolenic acid; values in the table refers to ranges in the percentage composition of individual fatty acids in the studies cited

### Discussion

Diversifying natural plant diets (inflorescences of grasses) cannot alter the content and compositions of the most common fatty acids in *R. differens* when reared throughout the entire life-cycle (although the composition of rare fatty acids was altered). The rationale for this might be that when *R. differens* feed on the natural diet, they produce fatty acids through de novo biosynthesis. However, the composition of fatty acids in the wild-harvested sixth instar nymphs of *R. differens* differed significantly when reared for 2 weeks on different mixtures of its natural plants (inflorescences of grasses) [10]. The reason why differences in fatty acid content and composition did not emerge here, when *R. differens* are reared throughout their life-cycle, could be due to the conversion of accumulated fatty acids to other biosynthetic precursors and utilization for other body requirements during insect development [11, 12]. Linoleic and α-linolenic acids, for example, provide the building blocks for making arachidonic acid and eicosanoids [13]. Eicosanoids, though in limited proportions might be an indication of its role in immune defensive mechanisms and reproduction of *R. differens*, as for various insect species [12–14]. The fatty acid profiles in the tissues of insects can change drastically after neonate nymphs’ metamorphosise through developmental stages into maturity [14].

The most common fatty acids found included palmitic, stearic, oleic, linoleic and α-linolenic acids. With exception of α-linolenic acid (12–16%), other common fatty acids (Table 3) had relatively similar proportions to those found in earlier studies [4, 6]. The contents of SFA and MUFA in *R. differens* were not altered by the diversifying natural food plant diets, suggesting that diets offered insects relatively similar SFA and MUFA contents. Fatty acids such as palmitic acid is used as precursors for the biosynthesis of long chain fatty acids [14]. The low content of SFA could suggest that certain MUFAs are synthesised from SFA precursors [14]. Biosynthesis of MUFA and SFA is a common phenomenon in many insect groups [14]. In comparison to [4] that reared neonate nymphs to maturity, the contents of SFA and MUFA in this study were generally low. Possibly the diet offered to *R. differens* in [4] was richer in SFA and MUFA compared to our grass inflorescences. When compared with insects analysed in [15], our samples had relatively low fatty acid content.

Our results show that the ratio of n − 6/n − 3 fatty acid is not altered by the natural plant diet representing inflorescences of grasses. However, the n − 6/n − 3 ratio in male *R. differens* was generally higher than in females possibly due to diverse physiological and metabolic functions between male and female or differences in utilization of these fatty acids [16]. For example, linoleic and linolenic acids are used in the ovarian development and egg production in females [12]. Overall, for both sexes, the n − 6/n − 3 fatty acid ratio was less than two, which is within the recommended range [13]. However, this n6/n3 ratio was low compared to previous studies for both reared [4], and wild-harvested *R. differens* [5, 6, 17].

Finally, our results showed that the weight of adult *R. differens* was not affected by the studied diets, corroborating our previous findings [10]. However, when fed with artificial diets manipulating fatty acid, carbohydrate and protein contents, differences in the weights emerged [4]. Many previous studies have indicated that the weight of an insect is largely determined by its diet and the development stage [18]. Therefore, it is likely that the fatty acids (or their precursors) were insufficient to build a heavy fat body with the grass inflorescence diets studied. Compared to our previous studies [19, 20], feeding on artificial diets generally produced *R. differens* with relatively higher weight (0.4–0.65 g) than with natural plant diets studied here (0.41–0.45 g), which could be related to a higher fat content of artificial diets than in grass inflorescences.

### Conclusion

The content and compositions of fatty acids in *R. differens* are not altered by diversifying grass inflorescences diets when reared throughout the entire lifecycle, except for the composition of rare fatty acids. The low n − 6/n − 3 fatty acid ratio as observed suggest that beneficial n − 6/n − 3 ratios for humans can be achieved by rearing edible insects on diversifying natural plant diet. Considering the low adult weight compared to previous studies, grass inflorescences alone may not be sufficient feed for *R. differens.*

## Limitation of the study

- Increasing the sample size would offer a better overview of the effect of diet diversification on the factors studied.

## Abbreviations

SFA: saturated fatty acid
MUFA: monounsaturated fatty acid
PUFA: polyunsaturated fatty acid
GC–FID: gas chromatography–flame ionization detector
ANOVA: analysis of variance
PERMANOVA: permutational multivariate analysis of variance
NMDS: non-metric multidimensional scaling
PERMDISP: permutational analysis of multivariate dispersion
